# Effect of oral contraceptive with and without associated estriol on ultrasound measurements of breast fibroadenoma: randomized clinical trial

**DOI:** 10.1590/S1516-31802007000500005

**Published:** 2007-09-02

**Authors:** Afonso Celso Pinto Nazário, Edmund Chada Baracat

**Keywords:** Fibroadenoma, Contraceptives, oral, Estriol, Ultrasonics, Growth substances, Fibroadenoma, Anticoncepcionais orais, Estriol, Ultra-som, Substâncias de crescimento

## Abstract

**CONTEXT AND OBJECTIVE:**

Fibroadenomas are the most common benign tumors of the female breast. The aim of this study was to evaluate the proliferative activity of breast fibroadenoma as shown by ultrasound measurements, following administration of oral contraceptives with and without associated estriol.

**DESIGN AND SETTING:**

This was a randomized, double-blind, placebo-controlled clinical trial carried out in the Mastology Sector, Department of Gynecology, Universidade Federal de São Paulo.

**METHODS:**

We studied 33 women with fibroadenomas. Ten were placed in group 1 and took an oral contraceptive consisting of levonorgestrel and ethinyl estradiol together with placebo material in the same capsule, for four consecutive cycles with a seven-day interval between them. The other 23 patients constituted group 2 and took the oral contraceptive as above together with estriol in the same capsule, in the same way as done by the group 1 patients. We took ultrasound measurements of their tumors (in three dimensions) before and after the intake of medication. At the end of the study, all the patients had their tumors removed by surgery.

**RESULTS:**

We observed decreased fibroadenoma width among the users of oral contraceptives with placebo, and this decrease was statistically significant. In the other group, we did not observe any changes (in width, length or height).

**CONCLUSION:**

The results confirm that estriol may block the protective effect of oral contraceptives on fibroadenomas, since we observed decreased fibroadenoma width among the group 1 patients but not the group 2 patients.

## RESUMO

**CONTEXTO E OBJETIVO:**

Fibroadenomas são os tumores benignos mais comuns na mama feminina. Avaliamos a atividade proliferativa do fibroadenoma mamário por medidas ultra-sonográficas após a administração de anticoncepcional hormonal combinado oral, associado ou não ao estriol.

**TIPO DE ESTUDO E LOCAL:**

Ensaio clínico randomizado, duplo-cego, placebo-controlado, realizado na Universidade Federal de São Paulo.

**MÉTODOS:**

Foram estudadas 33 pacientes portadoras de fibroadenoma, do setor de Mastologia da Disciplina de Ginecologia da Universidade Federal de São Paulo, sendo que 10 mulheres constituíram o grupo 1 e utilizaram anticoncepcional oral composto de levonorgestrel e etinilestradiol, associados a um comprimido de placebo, na mesma cápsula, por quatro ciclos consecutivos, com intervalo de sete dias entre os mesmos. As restantes 23 pacientes alocaram-se no grupo 2 e ingeriram, além do anticoncepcional oral descrito acima, um comprimido de estriol, que foi manufaturado conjuntamente ao anticoncepcional, em uma mesma cápsula, sendo utilizado da mesma forma que nas pacientes do grupo 1. Realizamos medidas ultra-sonográficas dos tumores (três dimensões) antes e após a ingestão da medicação. Ao término do estudo, as pacientes sofreram exérese de suas tumorações.

**RESULTADOS:**

Obtivemos diminuição da largura nos fibroadenomas de pacientes usuárias apenas de anticoncepcional oral e esse resultado foi estatisticamente significante. Não houve alteração de nenhuma dimensão (largura, altura ou comprimento) no outro grupo.

**CONCLUSÕES:**

Os resultados corroboraram que o estriol bloquearia o efeito protetor do anticoncepcional hormonal combinado oral sobre os fibroadenomas, já que observamos diminuição na largura dos fibroadenomas das pacientes do grupo 1 e não do grupo 2.

## INTRODUCTION

Fibroadenomas are the most common benign tumors of the female breast. This type of neoplasm consists of glandular and fibrous tissues and occurs in young patients, between the ages of 20 and 30 years. Fibroadenomas are the most common benign disease in females under the age of 35 and they occur without symptoms in 25% of the cases. They are multiple in 13 to 20%.^1–3^ In relation to race, they are more common among African Americans than within the white population.^4–6^ Fibroadenomas grow as well-limited and mobile spherical nodules and do not attach to neighboring breast tissue. They are most often located in the left breast and in the upper external quadrant. Their size ranges from small lesions of less than 1 cm to lesions of huge dimensions, such as 10 to 15 centimeters (giant fibroadenoma).^7^

Phyllodes tumor, fibrosarcoma, asymmetric juvenile giant breast, breast cysts, galactocele, hamartoma, adipose necrosis, hematoma and breast cancer are the possible differential diagnoses.^1^

The treatment for breast fibroadenoma is heterogeneous. Tumors of less than one centimeter may undergo partial or total regression in up to one third of the cases.^8^ A wait-and-see approach may be the best option, particularly for small tumors in females under the age of 25 years, or under the age of 35 years without familial risk of breast cancer.^9^ In other cases, surgical resection may be considered.

Estrogens seem to be linked to fibroadenoma genesis.^10^ Fibroadenomas occur mainly in young females who frequently choose oral contraceptives as a contraceptive method. Oral contraceptives contain synthetic estrogens and progestogens in their formulae. The estrogen most used in oral contraceptives is ethinyl estradiol, which has oral bioavailability because of the addition of an ethyl group in the 17^th^ position of estradiol. The doses of ethinyl estradiol used today are lower than in the past, and now range from 15 to 30 μg.^11^ The progestogen component varies in a great number of synthetic compounds and acts like natural progesterone. Progestogens are differentiated according to their power to reproduce progestogenic effects. The first generation of progestogens includes norethindrone and ethynodiol diacetate; the second generation is represented by the levonorgestrel and norgestrel and the third by desogestrel and norgestimate. One of the newest progestogens is drospirenone, derived from 17-alpha-spironolactone, which has antimineralocorticoid and antiandrogenic effects.^12,13^

Oral contraceptives inhibit ovulation by blocking the production and liberation of follicle-stimulating hormone (FSH) and luteinizing hormone (LH). The suppressive effect of oral contraceptives on gonadotrophin production depends on synergic action by the estrogenic and progestogenic compounds in the pills. This action changes the amplitude of gonadotropin-releasing hormone (GnRH) pulses and has a direct suppressive effect upon the hypophysis, thereby inhibiting the LH apex and affecting the endocrine mechanisms of ovulation. With suppression of ovulation, the luteal body is not formed and does not produce progesterone.^14–16^ Moreover, oral contraceptives act on other sites of the reproductive system. At the uterine coli they change the mucus, making it thick and hostile to sperm migration. They lessen the production of glycogen in endometrial glands, thus inhibiting their proliferation and maturation, which complicates nidation. They also alter the motility of the uterine tubes, thereby interfering with ovule transport.^13^

In fact, the inhibition of fibroadenoma growth by means of oral contraceptives or hormone replacement therapy is still a controversial topic. While Ravnihar et al.^17^ (1979) demonstrated that hormone therapy was a protection factor, Sitruk-Ware et al.^18^ (1989) does not observe such an influence.

Therefore, we proposed to evaluate the effect of oral contraceptive use on breast fibroadenoma behavior, through the use of breast ultrasound. We were also interested in investigating whether any drug used in conjunction with the oral contraceptive might influence the cellular kinetics of this neoplasm. We suspected that estriol might inhibit the oral contraceptive effect on fibroadenomas. Estriol is, in fact, the peripheral metabolite of estrone and estradiol, not a secretory product from the ovaries. Its formation occurs because of metabolic detoxification, i.e. the conversion of active substances into less active forms.^19^

## OBJECTIVE

In a review of the literature, we did not find any studies evaluating the effects of estriol, with or without oral contraceptive, on fibroadenomas. Therefore, we decided to administer estriol together with the oral contraceptive, in order to investigate its behavior in relation to breast fibroadenoma, through ultrasound measurements.

## MATERIAL AND METHODS

We selected 70 women initially, but only 33 reached the inclusion criteria of our study. These women attended consultations at the Mastology outpatient clinic for benign mammary disorders of the Gynecology Department, Universidade Federal de São Paulo, between March 2001 and January 2005. The inclusion criteria for the patients were that they had to be healthy women with benign breast tumors diagnosed by clinical, cytological and radiological evaluations, with ages ranging from 19 to 35 years. They had to present regular menstruation over the past six months and had to know when their last menstrual period occurred. Their gynecological and colposcopic examinations had to present normal results. We excluded patients presenting endocrine disorders, pregnant women, patients in the puerperium stage and patients with suspected carcinoma.

The project was analyzed and approved by the Research Ethics Committee of Universidade Federal de São Paulo.

All the patients underwent clinical examination, including anamnesis, physical examination, fine-needle aspiration biopsy, oncological-cytological tests, ultrasound and, when indicated, mammography. During the ultrasound examination, we took measurements of the tumor length, width and height. This was done both before and after the patient took the medication. These measurements were recorded in a protocol file, along with the other information mentioned above. The measurements that were taken before and after treatment were subjected to statistical analysis using Student's t test^20^ and the non-parametric Wilcoxon test.^21^ We took a significance level of 5% for all the tests performed.

The 33 patients selected for this study were divided into two groups: group 1 (control) with 10 women with delimited fibroadenomas who used oral contraceptive consisting of levonorgestrel (0.15 mg) and ethinyl estradiol (0.03 mg) together with placebo material inside the same capsule, for up to four consecutive cycles with a seven-day interval between them. Group 2 was constituted by another 23 patients with benign breast tumors who took the same oral contraceptive, but with 2 mg of estriol instead of the placebo, inside the same capsule, for up to four consecutive cycles. At the end of the study, all the patients had their tumors removed surgically.

In all cases, we measured the serum progesterone levels by an immunoassay technique on the day of the biopsy in order to ascertain the anovulatory effect of the oral contraceptive.

The study was conducted in a double-blind manner. We lost 37 patients during the study for a variety of reasons, and these were all patients in group 1. This was why there were differences in patient numbers between the two groups used in this study, since the divisions were only known after all the material was collected, i.e. after the tumor biopsies, during the statistical analysis.

## RESULTS

We did not observe any great disparity in the variables studied (age at menarche, number of gestations, parity, number of abortions and use of breastfeeding). Therefore, we considered that the groups were homogeneous, as shown in Table 1.

**Table 1.** Epidemiological data on the study patients, by groups

Patients	Age (years)	Age at menarche (years)	Number of gestations	Parity	Number of abortions	Duration of lactation (months)
**Group 1**						
IS	25	14	0	0	0	0
RAC	17	11	0	0	0	0
EAP	18	13	0	0	0	0
MCN	22	11	1	1	0	0
PCJ	29	11	0	0	0	0
VCAJ	18	13	0	0	0	0
VSN	19	13	0	0	0	0
MPSS	20	12	0	0	0	0
ACS	23	11	0	0	0	0
IVSS	18	12	0	0	0	0
** Mean**	**20.9**	**12.1**				
**Group 2**						
MMC	29	12	0	0	0	0
PPS	20	10	0	0	0	0
CAS	25	13	1	1	0	30
LSA	23	12	0	0	0	0
ACCM	15	11	1	0	1	0
MAL	20	14	0	0	0	0
RNS	34	16	3	3	0	36
RPP	33	15	0	0	0	0
SRA	21	13	0	0	0	0
ROR	18	12	0	0	0	0
FLR	24	13	5	5	0	0
RGLN	26	13	1	1	0	48
HSZ	18	13	0	0	0	0
SMS	18	13	0	0	0	0
NRG	14	9	0	0	0	0
SSS	30	12	3	2	1	26
RALS	30	10	2	2	0	24
SLOC	16	12	0	0	0	0
RMN	17	12	0	0	0	0
DT	24	13	1	0	1	0
JBS	23	15	0	0	0	0
RMM	29	13	0	0	0	0
JMPS	20	15	0	0	0	0
** Mean**	**22.9**	**12.7**				

Tables 2 and 3 show the ultrasound measurements on the patients’ breast tumors during the study groups 1 and 2. We took measurements of length, width and height before and after the patients took the medication. Progesterone serum levels were also measured to confirm that the patients were in a state of anovulation on the day of the tumor biopsy.

**Table 2.** Ultrasound measurements on tumors (mm) before and after medication and progesterone serum levels after medication in patients with fibroadenomas who received oral pills with placebo (group 1)

Patients	Measurements before intake (mm)	Measurements after intake (mm)	Progesterone (ng/ml)
IS	18 × 11 ×10	23 × 9 × 9	0.5
RAC	14 × 12 × 14	14 × 11 × 11	1.4
EAP	17×9×15	18 × 11 × 15	0.3
MCN	36 × 35 × 16	33 × 32 × 14	0.5
PCJ	13 × 8 × 6	13 × 6 × 5	0.3
VCAJ	30 × 15 × 24	30 × 16 × 25	0.4
VSN	24 × 18 × 21	25 × 14 × 23	< 0.2
MPSS	20 × 16 × 20	18 × 12 × 17	< 0.2
ACS	23 × 16 × 20	28 × 15 × 24	0.3
IVSS	21 × 17 × 13	22 × 12 × 20	0.3

**Table 3.** Ultrasound measurements on tumors (mm) before and after medication and progesterone serum levels after medication in patients with fibroadenomas who received oral contraceptives with estriol (group 2)

Patients	Measurements before intake (mm)	Measurements after intake (mm)	Progesterone (ng/ml)
MMC	28 × 26 × 13	28 × 14× 27	0.3
PPS	23 × 12×9	22 × 16× 10	0.8
CAS	28 × 27 × 11	32 × 14×11	0.3
LSA	12 × 9 × 9	11 × 6 × 8	0.2
ACCM	38 × 22 × 30	40 × 21 × 28	0.3
MAL	24 × 10 × 18	20×9×19	0.5
RNS	40 × 19 × 33	41 × 16 × 34	0.4
RPP	17 × 10 × 17	20 ×10× 19	< 0.2
SRA	24 × 16 × 18	25× 16×20	0.9
ROR	15 × 14 × 9	14 × 13 × 8	< 0.2
FLR	20 × 9 × 20	20 × 9 × 20	12.6
RGLN	28 × 12 × 25	29 × 22 × 12	1.6
HSZ	20× 8 ×12	18 × 8 × 13	0.5
SMS	60 × 15 × 60	55 × 16 × 51	0.2
NRG	27 × 21 × 25	27 × 23 × 26	0.3
SSS	21 × 16 × 18	22 ×16× 17	0.4
RALS	33 × 19 × 28	32 × 17× 27	0.3
SLOC	19 × 15 × 10	22 × 13 × 21	0.3
RMN	21 × 10 × 18	24× 12 ×15	0.4
DT	22 × 11 × 19	25 × 12 × 17	0.4
JBS	23 × 10 × 22	28×9×27	0.5
RMM	22 × 15 × 17	23 × 15 × 19	0.3
JMPS	29 × 9 × 20	25 × 12 × 18	0.4

Table 4 demonstrates the statistically significant different results from the fibroadenoma width measurements on the patients who took oral contraceptives with placebo (group 1; hatched cell in table) before and after treatment. This difference was not seen among the patients who took oral contraceptives with estriol (group 2). This result was confirmed by Student's t test and the Wilcoxon test. With regard to other measurements, there were no statistically significant results from either of the groups studied.

**Table 4.** Means and standard errors of ultrasound measurements on fibroadenoma tumors (mm) before and after medication for groups 1 and 2

Group 1 Oral contraceptive with placebo	Before medication		After medication		Descriptive level	
	Mean (mm)	Standard error	Mean (mm)	Standard error	Student's † test	Wilcoxon Test
Length	21.66	2.24	20.05	2.84	0.364	0.575
Width	15.73	2.40	13.17	2.63	0.043	0.036
Height	15.94	1.74	15.67	2.62	0.854	1.000
**Group 2 Oral contraceptive with estriol**	**Before medication**		**After medication**		**Descriptive level**	
	**Mean (mm)**	**Standard error**	**Mean (mm)**	**Standard error**	**Student's † test**	**Wilcoxon Test**
Length	25.10	2.22	26.44	1.89	0.158	0.122
Width	15.30	1.23	13.76	1.08	0.157	0.146
Height	20.79	2.47	20.93	2.30	0.913	0.926

## DISCUSSION

Most women want to control their own fertility. Every year, more than half a million women die for pregnancy-related reasons. Many of these deaths occur following an undesired pregnancy and are the result from 20 to 40% of the abortions that were performed inadequately. Oral contraceptive use may lessen the risks originating from pregnancy, births and abortions that are performed under inappropriate conditions.^22^

Oral contraceptives act on several organs with proven beneficial effects, such as: control of the menstrual cycle;^13,22^ prevention of ectopic pregnancy;^13,22^ control over acne and hirsutism;^13^ protection against pelvic inflammatory disease;^13^ reduction of the risk of ovary cancer;^23^ reduction of the risk of endometrial cancer;^24^ reduction of the incidence of benign breast disorders.^13,25^ However, in addition to the benefits, oral contraceptives may cause collateral effects such as menstrual irregularity, sickness and in some cases weight gain, breast pain and headaches.^13^ The worst risks through the use of oral contraceptives relate to the cardiovascular system, such as thromboembolism, arterial hypertension, vascular cerebral accident and heart attacks.

In relation to benign breast disorders, oral contraceptives provide decreases of up to 50 to 75% in the risk of fibroadenoma, cystic alterations and breast pain.^25,26^ However, the mechanisms through which oral contraceptives act to decrease the incidence of these conditions are still unclear.

Sawhney et al.^27^ (1992) studied epithelium and stroma in fibroadenomas and phyllodes tumors and demonstrated a spatial relationship between stroma mitosis and epithelial tissue concentration. They measured epithelial distribution in successive concentric rings surrounding fibroblast mitoses. They observed that when the distance between the stroma and epithelium was more than 200 μm, the stromal mitotic activity was expandable because it was limited to the spread of oxygen between stroma and epithelium. This distance represents the limit to the reach of passive oxygen diffusion, and it proves that fibroadenoma proliferation depends on local humoral factors of paracrine nature and not on endocrine mechanisms. Thus, there were some stromal regions with greater proliferative activity and higher epithelium concentration, while the mitotic activity of fibroblasts was lower in other stromal regions where the epithelium was distant.

Hasebe et al.^28^ (1999) studied standard fibroadenomas with low stromal activity using analyses of PCNA expression (proliferative cell nuclear antigen). They also studied hypercellular fibroadenomas with higher activity of the stromal compartment and phyllodes tumors where the stromal activity was greatest. They found that the PCNA expression was low in standard fibroadenoma fibroblasts and that, as the stromal cellularity increased (as in hypercellular fibroadenomas and phyllodes tumors), there was increased PCNA expression. The greatest expression was in phyllodes tumors. Therefore, they suggested that stromal cellularity was regulated by the expression of fibroblast growth factor and by its receptors in paracrine growth pathways. Thus, fibroadenoma growth would depend on stromal compartment proliferation that was induced by growth factors produced in the epithelium.

In fibroepithelial tumors, the epithelial elements are inside a type of stroma that has an abnormal degree of proliferation but a uniform pattern. If stromal mitotic activity due to paracrine mechanisms depended on humoral factors produced by the epithelium, the proliferative activity would be expected to be as high, close to the epithelium, as was observed by Sawhney et al.^27^ These authors recognized that the epithelium had the capacity to produce growth factors like platelet-derived growth factor (PDGF), epidermal growth factor (EGF) and type 1 insulin-like growth factor (IGF-1), which act on fibroblasts to stimulate DNA synthesis and induce their growth.

These facts made some authors like Sawhney et al.^27^ (1992) and Pasqualini et al.^29^ (1997) accept the existence of local loop control such that the growth factors produced in the epithelium act in the stroma to make it proliferate. This stroma proliferation would produce new growth factors needed for epithelial growth, thereby giving rise to fibroadenomas. This interdependence is lost in malignant neoplasms, but it is still preserved in fibroepithelial tumors, which are specialized lesions of breast stroma with the capacity to stimulate growth of the neighboring epithelium, thereby reaching an equilibrium between growth and inhibition factors. In this way, the slow growth of fibroadenomas and the stabilization of their growth after reaching a certain size in a great number of patients can be explained.^6^ In small numbers of patients, this link is lost. Sarcomatous transformation may occur when the epithelium does not inhibit stromal growth, or carcinomatous transformation when the stroma does not block epithelial proliferation (by inhibiting growth factors).

**Figure 1:**
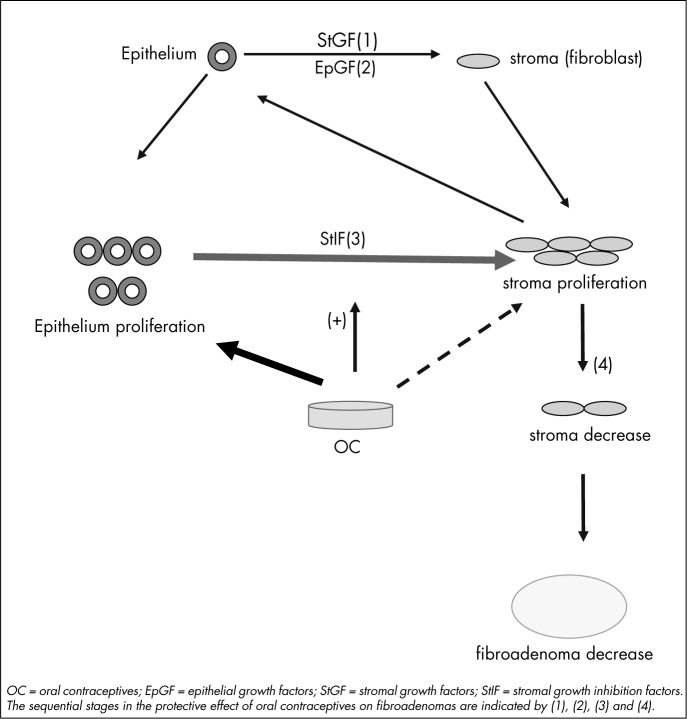
Model for paracrine control of fibroadenoma among oral contraceptive users.

The stronger action of ethinyl estradiol in oral contraceptives on epithelium causes it to proliferate and produce more stromal inhibitory factors. Through paracrine control, this causes less stroma proliferation. The final effect is decreased fibroadenoma dimensions (Figure 1) since the stromal compartment is responsible for fibroadenoma dimensions.^30,31^ This theory was confirmed by the findings of our study, in which we observed decreased fibroadenoma measurements by means of ultrasound, as shown in Table 2, among the patients who took oral contraceptives with placebo.

However, the influence of oral contraceptive on fibroadenomas is still questionable, since the studies in the literature are not uniform and the results are conflicting. Thus, some studies have demonstrated protective action through their use, such as the studies by Canny et al.^31^ (1988) and Rohan and Miller^32^ (1999), while in others, oral contraceptives caused increased incidence of fibroadenomas, such as in the study by Yu et al.^33^ (1992). Therefore, we put forward the hypothesis that estriol, as a weak estrogen, may competitively block the stronger actions of the ethinyl estradiol present in oral contraceptives. This allows us to indirectly demonstrate the important protective action of oral contraceptives on benign breast tumors, thus resolving the doubts that persist in the literature.

Our results indicate that the oral contraceptive use that decreased the fibroadenoma size among the patients in group 1 may represent a protective factor. However, this affirmation cannot be made from analysis of the results from group 2, i.e. the patients who took oral contraceptives with estriol. Among these patients, there were no variations in the ultrasound measurements of their tumors. This may have occurred because estriol was able to block the protective effect of the ethinyl estradiol in the oral contraceptives, as we will demonstrate below.

In fact, in group 1 (patients who took oral contraceptives with placebo) after four cycles of medication, we obtained a decrease in the ultrasound dimensions of their fibroadenomas and, more precisely, in their width. The difference in width was statistically significant, with a descriptive level of 0.043. We believe that the explanation for this finding is that, although ethinyl estradiol acts both on the stroma and on the epithelium of fibroadenomas, its effect occurs mainly on the epithelium, because this compartment has greater numbers of steroid receptors.^29^ Furthermore, in comparison with endogenous estradiol, ethinyl estradiol is much stronger. It stimulates the production of inhibitory growth factors by the epithelial compartment, which, through the paracrine control mechanism, acts on the stromal compartment to restrain its growth. Therefore, inhibition of stromal growth will take place through the epithelium of the fibroadenomas and, with extended use of oral contraceptives for four cycles, the stroma will gradually shrink and cause a decrease in tumor size, since this is the main compartment responsible for tumor dimensions^30^ (Figure 1). This was proven in our study by the decreased width of the fibroadenomas among the patients in group 1.

Estriol is an estrogen with weaker power, since it is a metabolic product.^34^ The possible selective antagonism between this and other powerful estrogens like estradiol and estrone, led us to use it in our study, in which it could competitively block the ethinyl estradiol found in oral contraceptives. We assumed that this antagonism would lead to a decrease in the protective effect of ethinyl estradiol on the epithelium of fibroadenomas.

The action mechanism for estriol works by competitive antagonism, i.e. by blocking estrogenic receptors and causing a small hormonal effect. Fibroadenoma epithelium has these receptors in greater numbers than does the stroma.^35^ Thus, we expected a greater epithelial block caused by estriol than was caused by the stroma. There might be less epithelial proliferation, which in turn would lead to decreased production of inhibition growth factors. Through the paracrine mechanism, the stroma would be less inhibited, which would leave it free to proliferate, thereby obstructing the overall decrease in fibroadenoma size. The absence of alteration in fibroadenoma measurements among the patients who took oral contraceptives with estriol (group 2) points in this direction.

On the other hand, stroma proliferation not only would maintain the tumor dimensions over the course of the cycles, but also could increase the production of growth factors that would act on the epithelium and thus lead it too to proliferate.

Therefore, it could be asked why in group 2 (patients who took oral contraceptives with estriol) we did not observe any decrease in fibroadenoma ultrasound dimensions in accordance with the hypothesis that estriol could competitively block the ethinyl estradiol, thereby obstructing its action on the epithelium. The explanation must be that the epithelium did not inhibit the stroma, which had its dimensions preserved, thus leading to unchanged ultrasound measurements in the final results (Tables 3 and 4).

At the end of this discussion, it must be taken into consideration that the small number of patients used in this study may put the conclusions at risk, since we obtained a weak discriminatory statistical power between the two groups.

## CONCLUSION

In conclusion, we obtained results that confirm that estriol may block the protective effect of oral contraceptives on fibroadenomas, since we observed clinical evidence of this through the decreased width of fibroadenomas among the patients in group 1 (oral contraceptives with placebo) and not in group 2 (oral contraceptives with estriol). However, we wish to emphasize that a study with a larger number of patients or a systematic review should be undertaken in order to obtain more conclusive results.
